# Complete Genome Sequence of an American Avian Leukosis Virus Subgroup J Isolate That Causes Hemangiomas and Myeloid Leukosis

**DOI:** 10.1128/genomeA.01586-14

**Published:** 2015-04-09

**Authors:** Sanandan Malhotra, James Justice, Nathan Lee, Yingying Li, Guillermo Zavala, Miguel Ruano, Robin Morgan, Karen Beemon

**Affiliations:** a Department of Biology, Johns Hopkins University, Baltimore, Maryland, USA; b Department of Population Health, College of Veterinary Medicine, University of Georgia, Athens, Georgia, USA; c Department of Animal and Food Sciences, University of Delaware, Newark, Delaware, USA; d Department of Biological Sciences, University of Delaware, Newark, Delaware, USA

## Abstract

We report the complete genome sequence of avian leukosis virus subgroup J (ALV-J) isolate PDRC-59831, which causes myeloid leukosis and hemangiomas in chickens. This is an American ALV-J isolate, which was found in a 38-week-old broiler breeder chicken on a farm in Georgia in 2007.

## GENOME ANNOUNCEMENT

The first ALV-J strain, HPRS-103, was isolated in the United Kingdom in 1989 from meat-type chickens with myeloid leukosis (ML) (1). ALV-J has caused huge economic losses to the poultry industry worldwide (2–6), resulting from drastically reduced egg production, stunted growth, bleeding tissues, and increased mortality (7, 8). ALV-J typically induces ML tumors, but some strains primarily induce hemangiomas (9). The isolate sequenced in this study induces both hemangiomas and ML tumors at high incidence**.** This new ALV-J isolate, designated PDRC-59831 (Poultry Diagnostic and Research Center, Athens, Georgia, USA) was isolated from a 38-week-old broiler breeder chicken.

We inoculated PDRC-59831 into 5-day-old SPAFAS embryos (Charles River) via the yolk sac route. All experimental chickens were euthanized when ill or by 12 weeks of age. Seven ALV-J infected chickens survived and were all found to have hemangiomas, ML, or both (10). The whole genome of PDRC-59831 was then amplified via PCR from genomic DNA of 4 different tissues from 3 infected birds. Sequence assembly and multiple sequence alignment were done using SnapGene and ClustalOmega.

Comparison of the PDRC-59831 sequence to the original English isolate, HPRS-103 (GenBank accession no. Z46390), showed that the *gag*, *pol*, and *env* sequences share a nucleotide identity of 97.2%, 97.7%, and 95.0%, respectively. In contrast, comparison to the other fully sequenced American isolate ADOL-7501 (accession no. AY027920), which also induces ML and hemangiomas in chickens (11), showed homologies of 95.1%, 97.2%, and 91.2% for these respective regions. We also observed the presence of upstream open reading frames (uORFs) in the RNA leader sequence that are known to modulate viral gene expression (12, 13). We found that, in comparison to the 3 uORFs found in RSV (GenBank accession no. J02342) and HPRS-103, PDRC-59831 has only 2 uORFs (uORF1 and uORF3).

Several studies reported certain genetic alterations in ALV-J strains that primarily induce hemangiomas in chickens. For example, an 11-nucleotide deletion was observed in the LTR-U3 region of ALV-J strains SCDY1 and NHH (14). Additionally, two different 19-nucleotide insertions in the 5′ untranslated region (5′ UTR) and in the U3 region were identified in the hemangioma-inducing strains JL093-1, SD09DP03, and HLJ09MDJ-1 (9). None of these sequence alterations were observed in PDRC-59831. Instead, PDRC-59831 has more sequence similarity to the ML-inducing prototype strain HPRS-103 in these regions. This suggests that these genetic alterations are not necessary to induce avian hemangiomas**.** Furthermore, a 205-nucleotide deletion in the 3′ UTR that leads to higher oncogenicity and increased mortality in infected chickens has also been identified in some ALV-J strains (15). Sequence alignment identified a similar deletion in PDRC-59831. This observed deletion of 216 nucleotides almost entirely encompasses the previously described 205-nucleotide deletion.

Our work provides additional understanding of the variations in ALV-J genomes, which can help determine evolutionary relationships among viral populations.

### Nucleotide sequence accession number.

The complete genome sequence of the PDRC-59831 isolate was submitted to GenBank under the accession number KP284572. The same viral isolate was used for characterizing ALV-J common integration sites in the chicken genome (10).
